# QTL-mapping and genomic prediction for bovine respiratory disease in U.S. Holsteins using sequence imputation and feature selection

**DOI:** 10.1186/s12864-019-5941-5

**Published:** 2019-07-05

**Authors:** Jesse L. Hoff, Jared E. Decker, Robert D. Schnabel, Christopher M. Seabury, Holly L. Neibergs, Jeremy F. Taylor

**Affiliations:** 1 Division of Animal Sciences, University of Missouri, Columbia, MO 65211 USA; 2 Informatics Institute, University of Missouri, Columbia, MO 65211 USA; 3 Department of Veterinary Pathobiology, Texas A&M University, College Station, TX 77843 USA; 4 Department of Animal Sciences, Washington State University, Pullman, WA 99163 USA

**Keywords:** Genomic prediction, Bovine respiratory disease, Genome sequence imputation, RNA-Seq, Quantitative trait locus, Feature selection

## Abstract

**Background:**

National genetic evaluations for disease resistance do not exist, precluding the genetic improvement of cattle for these traits. We imputed BovineHD genotypes to whole genome sequence for 2703 Holsteins that were cases or controls for Bovine Respiratory Disease and sampled from either California or New Mexico to construct and compare genomic prediction models. The sequence variation reference dataset comprised variants called for 1578 animals from Run 5 of the 1000 Bull Genomes Project, including 450 Holsteins and 29 animals sequenced from this study population. Genotypes for 9,282,726 variants with minor allele frequencies ≥5% were imputed and used to obtain genomic predictions in GEMMA using a Bayesian Sparse Linear Mixed Model.

**Results:**

Variation explained by markers increased from 13.6% using BovineHD data to 14.4% using imputed whole genome sequence data and the resolution of genomic regions detected as harbouring QTL substantially increased. Explained variation in the analysis of the combined California and New Mexico data was less than when data for each state were separately analysed and the estimated genetic correlation between risk of Bovine Respiratory Disease in California and New Mexico Holsteins was − 0.36. Consequently, genomic predictions trained using the data from one state did not accurately predict disease risk in the other state. To determine if a prediction model could be developed with utility in both states, we selected variants within genomic regions harbouring: 1) genes involved in the normal immune response to infection by pathogens responsible for Bovine Respiratory Disease detected by RNA-Seq analysis, and/or 2) QTL identified in the association analysis of the imputed sequence variants. The model based on QTL selected variants is biased but when trained in one state generated BRD risk predictions with positive accuracies in the other state.

**Conclusions:**

We demonstrate the utility of sequence-based and biology-driven model development for genomic selection. Disease phenotypes cannot be routinely recorded in most livestock species and the observed phenotypes may vary in their genomic architecture due to variation in the pathogen composition across environments. Elucidation of trait biology and genetic architecture may guide the development of prediction models with utility across breeds and environments.

**Electronic supplementary material:**

The online version of this article (10.1186/s12864-019-5941-5) contains supplementary material, which is available to authorized users.

## Background

### Economic cost

Bovine respiratory disease (BRD) impacts the U.S. cattle population more than any other communicable disease. In a recent study, the estimated economic cost of a BRD case was $253.97 [1]. Management tools to limit the impacts of BRD are limited due to the broad array of responsible pathogens and the lack of effective vaccines [2]. Furthermore, diagnosis of the pathogens responsible for disease in affected animals is uncommon and has prevented the development of predictions of genetic merit for disease risk and their incorporation into selection indexes [3].

### Previous study

To develop genetic resources for the improvement of resistance to BRD, a case-control study was performed in 2703 Holstein calves from New Mexico (NM) or California (CA) [4]. Phenotypes were collected using the McGuirk scoring system [2] and animals were genotyped with the Illumina BovineHD (HD) assay to characterize the genetic architecture of resistance to the underlying pathogens responsible for disease and to identify large-effect QTL. Disease traits such as BRD are difficult to develop prediction models for the implementation of Genomic Selection due to the lack of phenotype data. Consequently, the greatest opportunity for the development of genomic predictions (GP) for risk of BRD industry-wide is to leverage developed resource populations to generate GP with utility across environments and potentially also across breeds.

An important observation made in the initial study was that there appeared to be substantial genetic heterogeneity between environments for risk of BRD [4]. While moderate estimates of percent variation explained (PVE) on the observed scale (21–22%) for risk of BRD were obtained in the analyses performed within each state, when the data for the two states were combined, the PVE estimate substantially decreased (~ 14%). This was interpreted to be a consequence of genetic heterogeneity in the immune response due to the presence of different pathogen profiles present within each environment as established from pathogen diagnostic analyses of deep nasopharyngeal and mid-nasal swabs taken from each sampled animal.

### Goals of this study

QTL mapping and GP for risk of BRD require approaches that differ from the neutral marker paradigm that has successfully been applied to routinely recorded livestock production traits [5]. Genetic heterogeneity between populations occurs when sick animals display the same clinical signs, but these signs are caused by infections involving different pathogens that elicit potentially different immune responses. When this occurs, mutations in different genes and regulatory elements may lead to susceptibility to different pathogens which also differ in their prevalence across environments, leading to an apparent genotype-by-environment interaction for risk of BRD. When this is the case, the genetic correlation between risk of BRD in the different environments will be substantially less than one and integrating data for animals from different environments will result in the identification of QTL or production of GP that have a reduced pathogen specificity. This approach will therefore tend to reduce the ability to identify variants with pathogen-specific effects on risk of BRD. Considering the cost and difficulty of creating GP training populations representing the range of U.S. cattle production environments, it is imperative that we fully leverage existing disease status data. We attempted to utilize different data types to enhance our biological understanding of BRD and better understand the genetic architecture of disease risk within and across regions.

We utilized two strategies in an attempt to improve the utility of GPs across geographic regions of the U.S. using the data reported in Neibergs et al. [4]. The first approach involved the imputation of the HD genotype data to the level of whole genome sequence (WGS) variation using a large reference panel of sequenced individuals from Run 5 of the 1000 Bull Genomes Project (1KBGP) [6]. In the second approach, we examined the effects of reducing the number of single nucleotide polymorphisms (SNPs) from the WGS imputation set that were included in the GP model to sets selected based upon a genome-wide association (GWAS) analysis of these data and a previous RNA-Seq analysis. We included SNPs located within genomic regions found to harbour QTL of large effect on risk of BRD, or SNPs located within genomic regions previously found to harbour genes involved in the normal immune response to infection by pathogens responsible for BRD based on RNA-Seq analysis [7] and the union of these two sets of variants. Recent studies have suggested the utility of incorporating ancillary information into the analysis to inform the selection of biologically relevant loci and reduce the challenge of estimating effects for several hundreds of thousands, or millions, of loci [8–11].

When the genetic architecture of a trait varies across environments, we postulate that improved predictions could be produced by enriching the prediction model for SNPs that tag segregating disease risk QTL detected in multiple environments or variants associated with immune response loci. The first approach uses a two-step data analysis in which the first phase estimates WGS variant effects and the second, feature selection step, retains only those SNPs that are located in large-effect QTL regions for the prediction of genetic merit. We recognise that this does not represent the use of independent data to identify QTL and that the estimated QTL effects are positively biased, which is commonly known as the “Beavis effect” (see [12] for an explanation). Consequently, the selection of SNPs in QTL regions detected in an analysis of the same data set is likely to result in a positive bias in the accuracy of GPs. We sought to quantify the magnitude of this bias by performing cross-validation analyses in which subsets of the data were used for QTL detection and the training of the prediction model which was then applied to an independent subset of the data for validation of prediction accuracy. The second approach utilized completely independent biological information to identify genomic regions harbouring genes involved in the normal immune response to infection by pathogens causing BRD and then selecting SNPs from the WGS variants located in these regions for the prediction of genetic merit.

Our objectives were to examine the utility of these analytical frameworks for developing a single GP model for risk of BRD across geographic regions of the U.S. and for improving our biological understanding of the mechanisms of disease resistance in livestock via more precise QTL mapping using WGS imputed variants.

## Results

### Imputation

**Fig. 1:**
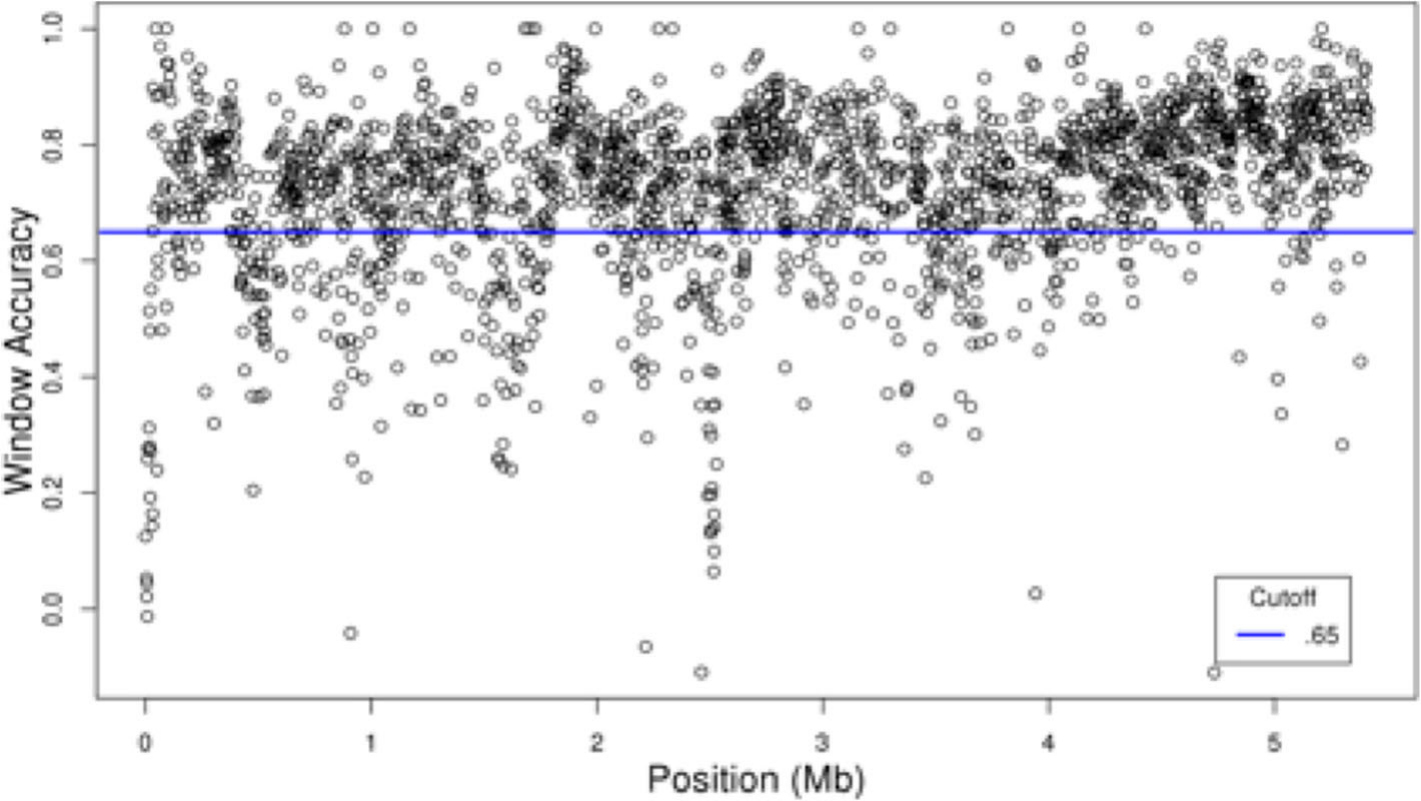
A window-based analysis of SNP genotype imputation accuracy performed with Genwin for chromosome 23. By fitting splines to individual locus imputation accuracies within 10-kb windows and pooling windows with similar accuracies, regions with low imputation accuracy can be identified

Imputation resulted in 39,721,988 SNPs with genotypes predicted in the 2703 animals. To estimate the accuracy of imputation, we performed a separate imputation analysis using only the 1KBGP Run 5 reference set. Here, we excluded the genotype data for the 29 Holsteins sequenced from among our BRD study animals from the WGS reference set and produced a direct measure of imputation accuracy as the Pearson correlation between their WGS imputed and WGS called genotypes. Overall mean per SNP genotype imputation accuracy was 76%. However, we observed regions throughout the genome where genotype imputation accuracy was very low (Fig. 1). The boundaries for these regions were estimated using a spline fitting analysis of imputation accuracy correlations in 10-kb windows throughout the genome using GenWin [13]. Imputed variants were then filtered either on the basis of their individual SNP imputation accuracy or regional accuracy using the regions of low (< 65%) average imputation accuracy identified by GenWin. We also filtered all variants with a minor allele frequency < 5%. This resulted in a set of 9,282,726 imputed variants for the 2703 animals for which the average imputation accuracy was estimated to be 84.2%.

### Association analyses

**Fig. 2:**
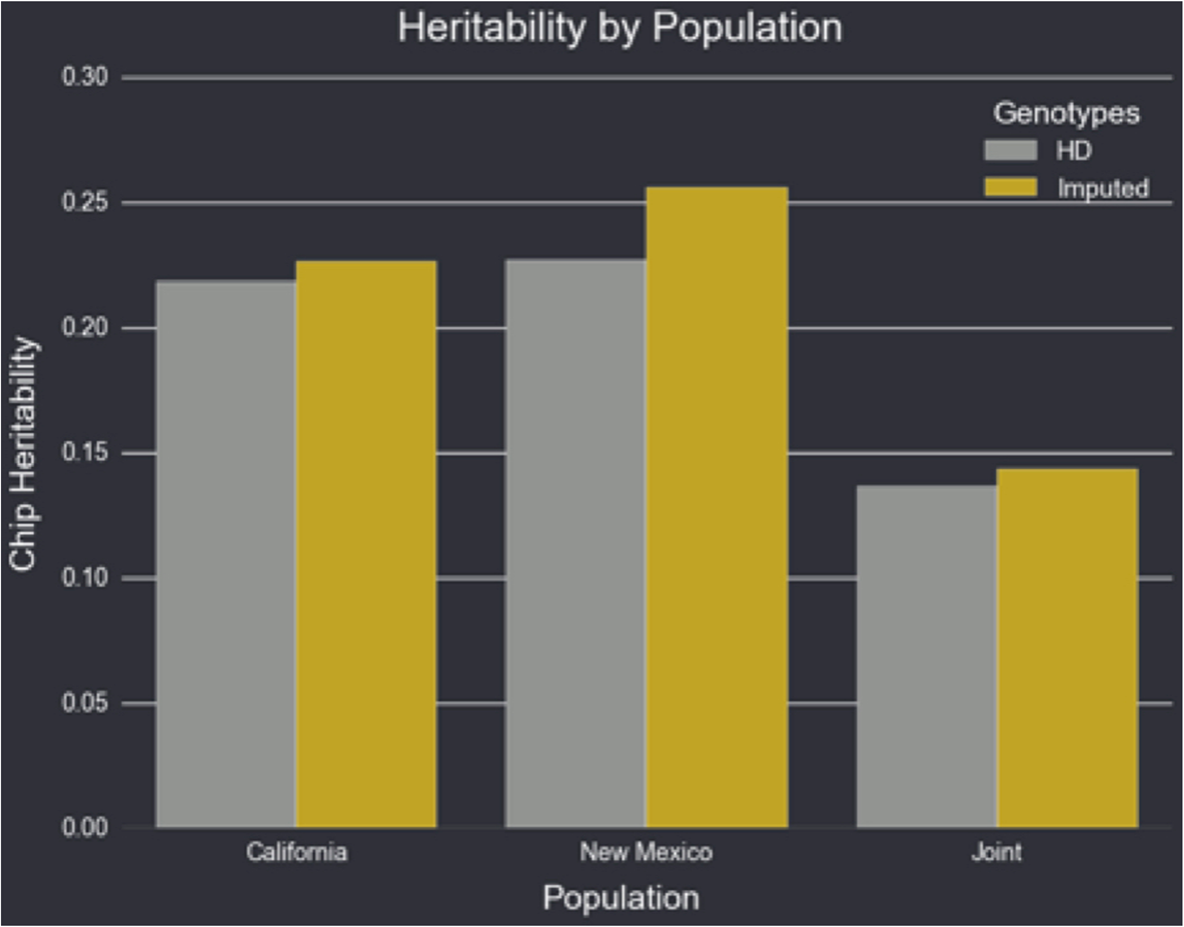
Estimates of the proportion of phenotypic variance explained by marker genotypes for the BovineHD assay or whole genome sequence imputed genotypes for BRD as a case-control phenotype by state and across states

**Fig. 3:**
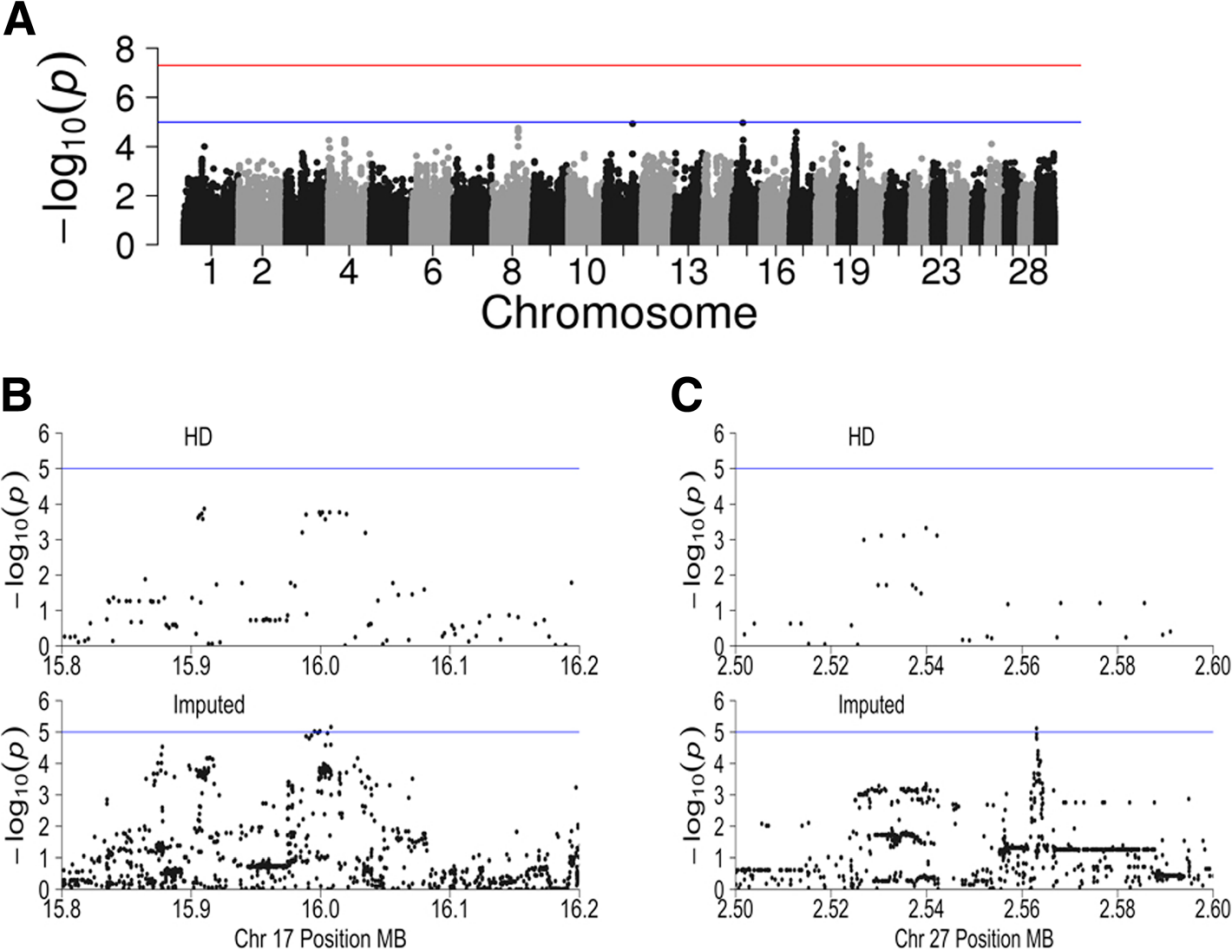
GWAS analysis of the combined CA + NM cohorts. A) Genome-wide, the imputed WGS data reveal stronger associations than found by Neibergs et al. [4]. In some cases, this involved the strengthening of signal at QTL detected in the analysis of the BovineHD data, such as in panel (B) containing *INPP4B* (chr17:15,338,071-16,116,053 bp) which is involved in T-cell differentiation and poly-phosphatase signaling and was found to be differentially expressed in the challenge experiment bronchial lymph node RNA-Seq data (Tizioto et al. [7]). In other cases, novel regions were revealed such as in panel (C) which shows a region partially containing *CSMD1* (chr27:1,016,499-2,688,276 bp), which regulates the complement system controlling inflammatory responses

**Fig. 4:**
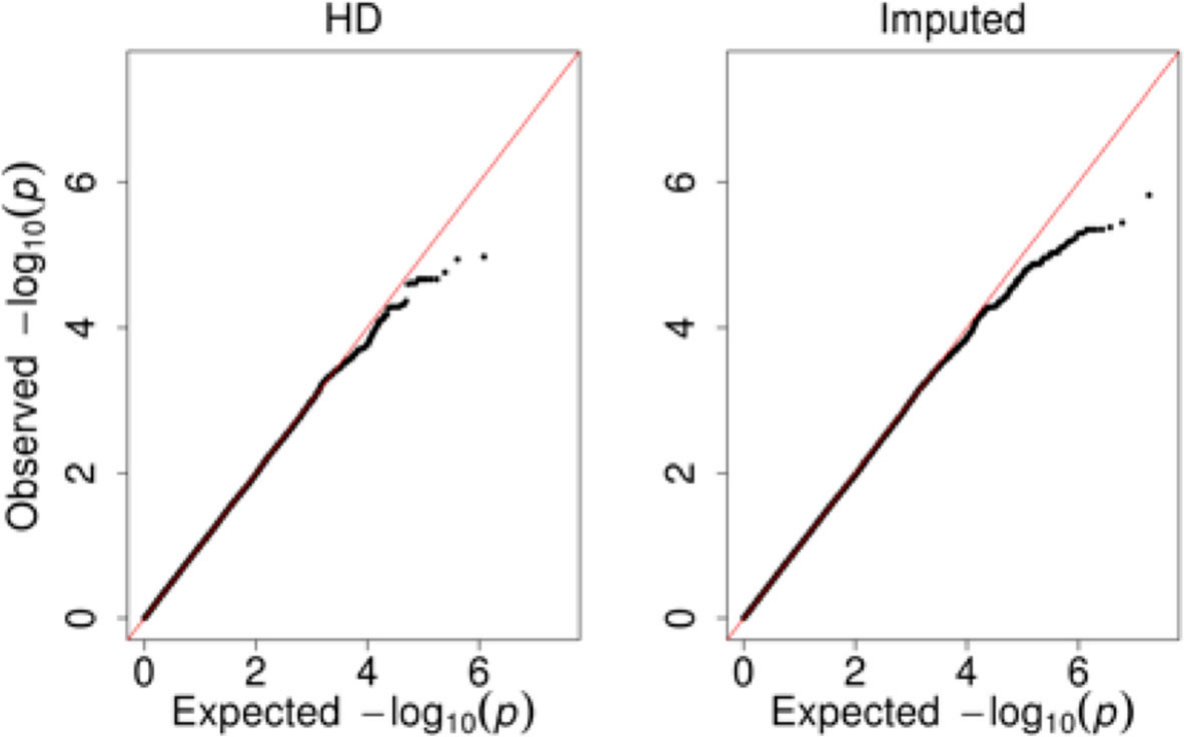
QQ Plots for the GWAS of the combined CA + NM cohorts. Left panel is for the analysis of the case-control data from the BovineHD data and right panel is for the imputed WGS data

Results of the association analyses of the imputed WGS data differed from those for the HD analyses in two ways. The estimates of PVE improved and mapping signals refined the locations of detected QTL. PVE estimates within each state increased (Fig. 2). However, the decrease in the estimate of PVE remained when the CA and NM cohorts were combined [4] and when the case-control data for the two states were analysed as separate traits in a bivariate model [14] implemented in GCTA, the genetic correlation between risk of BRD in CA Holsteins and NM Holsteins was estimated to be − 0.36. Imputed variants with strong association signals were found both within QTL regions previously detected in the analyses of the HD data [4] and in novel regions that were only detected in the analysis of the imputed WGS variants (Fig. 3). Genes such as *CSMD1*, which regulates the complement system controlling inflammatory responses are now implicated as candidates for variation in risk of BRD. The QQ-plots revealed only a small decline in the power of the imputed WGS genotype analysis relative to the HD analysis, despite the estimation of 14.2X more SNP effects (Fig. 4).

**Fig. 5:**
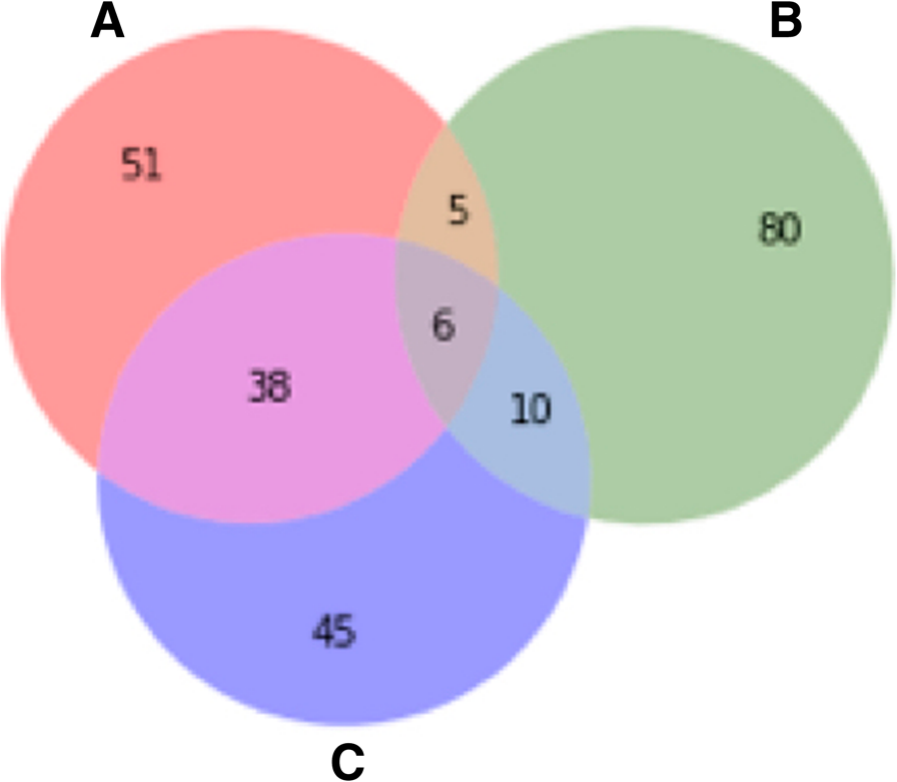
Overlap of 100 most significant QTL found in each of the 3 GWAS using imputed WGS data. **a**) CA, **b**) NM, and **c**) Joint analysis of CA and NM cohorts

**Fig. 6:**
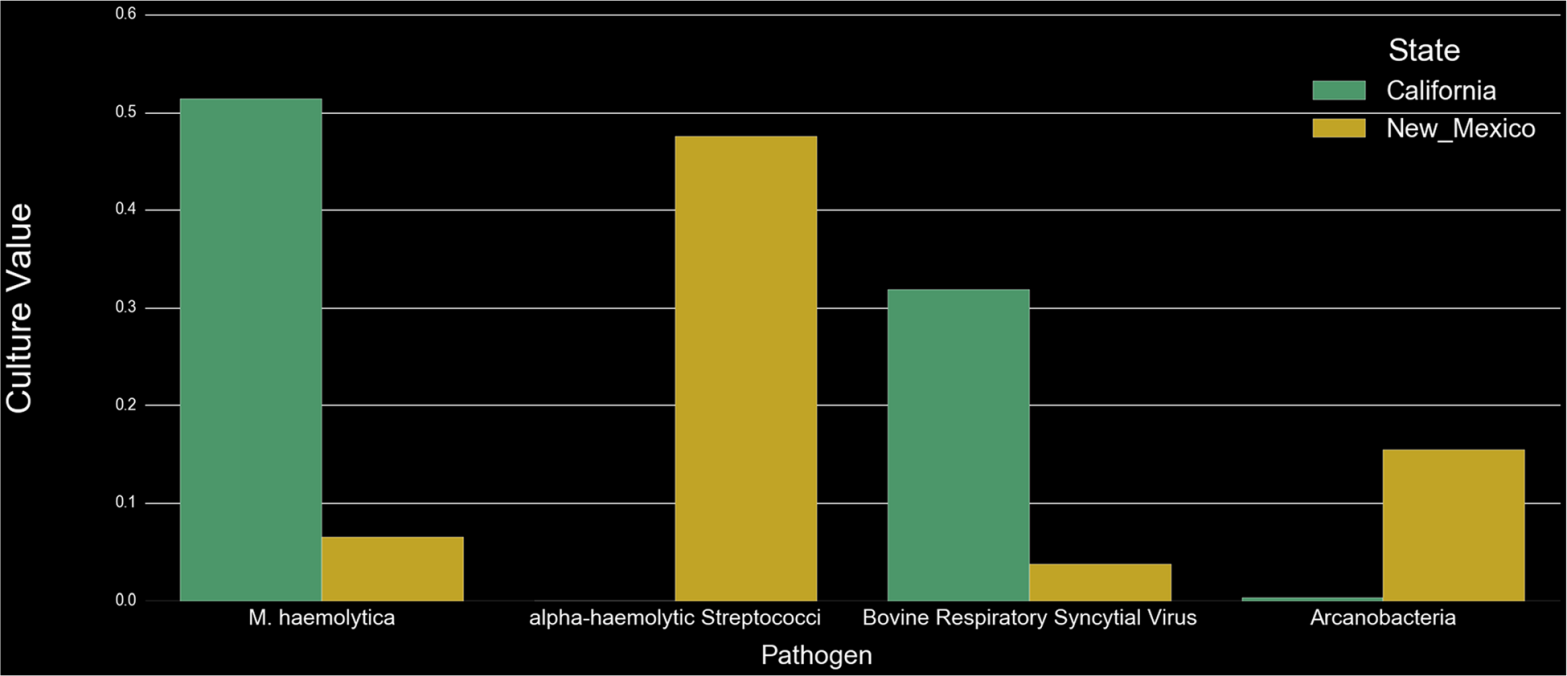
Pathogen prevalence determined from deep nasopharyngeal swabs taken from all cohort members in CA and NM for a subset of the detected pathogens

We also examined the regional overlap between QTL identified in the separate CA and NM analyses and observed that of the 100 most significant QTL found in each analysis, only 11 were common to both analyses (Fig. 5). Of these, only 6 QTL were found among the top 100 QTL in the joint analysis of the combined CA and NM data. Due to the larger CA sample size, more of the QTL identified in the analysis of the CA data (*N* = 44) than those identified in the analysis of the NM data (*N* = 16) were found among the 100 most significant QTL found in the analysis of the joint data. These results appear to be consistent with the finding that the pathogens detected in individuals from this study differed between the two regions (Fig. 6).

### Genomic predictions

**Fig. 7:**
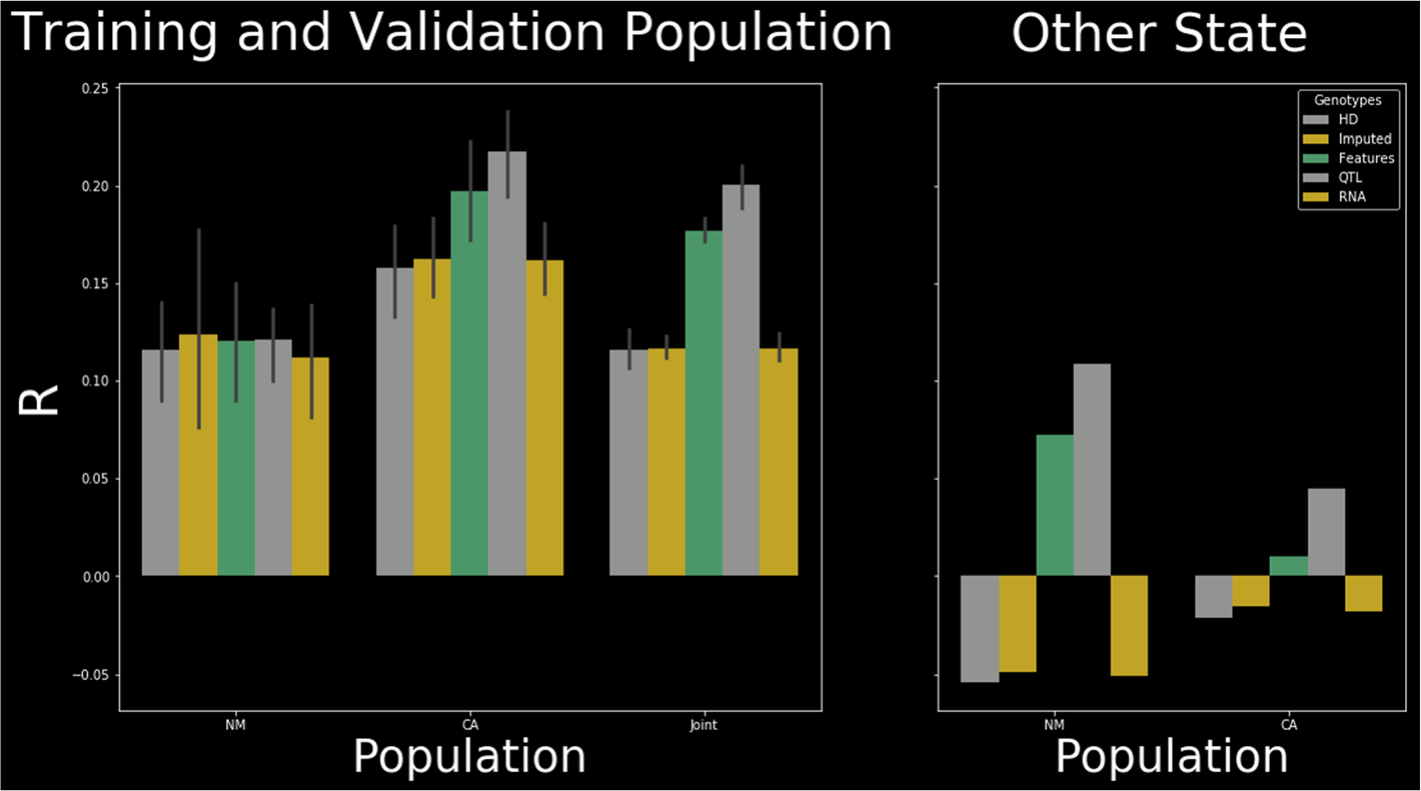
Validation sample correlations between genomic predictions trained using imputed WGS, BovineHD, QTL and RNA-Seq combined feature selection, QTL feature selection or RNA-Seq feature selection genotype data and BRD case-control phenotypes for different training and validation population designs. Left panel shows training and validation in samples of 50% of the data from each state or the combined CA and NM cohorts. Right panel shows validation correlations when training used all of the data for the indicated state and validation used the data for the other state

Five different training and validation scenarios were evaluated. Within each state and in the combined CA and NM cohorts, we performed two-way cross validations with five replicates. We also trained a model using the data for each state and then validated the prediction model accuracy using the data for the other state. These five separate training and validation analyses were run using both the HD and the imputed WGS data sets, resulting in ten comparisons for which results are reported in Fig. 7. For all fitted models, the accuracy of GPs was estimated as the Pearson correlation between the GPs and the observed BRD phenotypes. Accuracies and fitted regressions of GPs on phenotype are reported in Additional file 1: Table S1.

### Inconsistencies between the two states

**Fig. 8:**
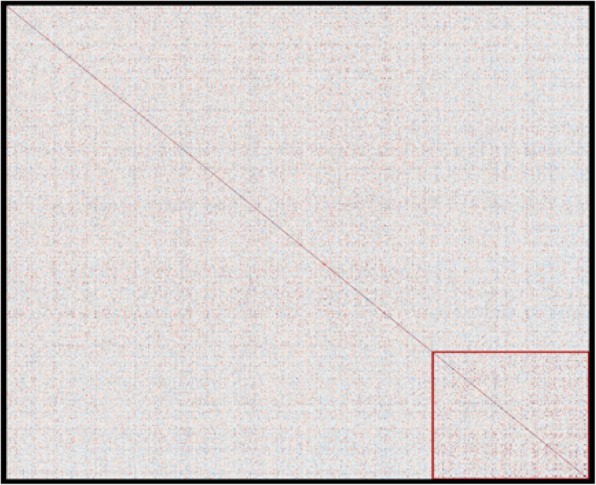
A heat map representation of the genomic relationship matrix constructed for all 2703 animals. NM animals are highlighted in the red box to the lower right

There were substantial differences in prediction model performance across the combinations of training and validation data sets. When training and validation occurred within the same state, prediction accuracy was in the range 0.112 ± 0.038 to 0.217 ± 0.030. Higher accuracies were achieved using the data from CA, undoubtedly due to the substantially larger sample size than for NM. However, when the data for CA and NM were combined, prediction accuracy declined. This result mirrors the change in estimated PVE from the association analyses, and occurred despite the increases in training data set size and marker density to WGS. When training the prediction model using the complete HD or WGS data for one state, the accuracies obtained when validating using the other state’s data were negative but close to zero consistent with the negative estimate of genetic correlation between risk of BRD in CA and NM. An analysis of the genomic relationship matrix (GRM) computed for all 2703 animals did not reveal any evidence for substructure between the sets of animals from the two states (Fig. 8). While the animals from NM had slightly higher levels of relatedness than those from CA, overall there was extensive shared ancestry between the animals from the two states.

### Accuracy of prediction models based on imputed WGS data

Consistent with the increase in PVE, the use of all imputed WGS genotypes to predict genomic breeding values for risk of BRD very slightly improved the accuracy of predictions over the use of the HD assay for the CA (HD: 0.158 ± 0.028 vs WGS: 0.162 ± 0.026), NM (HD: 0.116 ± 0.034 vs WGS: 0.123 ± 0.060) and Joint (HD: 0.116 ± 0.012 vs WGS: 0.117 ± 0.007) cohorts (Fig. 7), but the increases were not significant (*P* > 0.05).

### Feature selection

To examine if prediction accuracy could be improved, we implemented a feature selection approach to select variants for use in the creation of GP models. We first considered a two-stage analysis scheme in which GWAS was performed on the imputed WGS variants for the combined CA and NM cohorts and then the variants used for prediction were restricted to those within genomic regions putatively harbouring QTL. We next considered only the imputed WGS variants that were located in genomic regions harbouring genes previously identified as being involved in the normal immune response to infection by a pathogen responsible for BRD. Finally, we also utilized the 832,345 variants from the 9,282,726 imputed WGS variants that were in the union of these two feature sets.

#### QTL and RNA-Seq features

Models trained using the RNA-Seq feature selected variants performed similarly to the HD when trained and validated in the CA (RNA-Seq: 0.161 ± 0.023), NM (RNA-Seq: 0.112 ± 0.038) and Joint (RNA-Seq: 0.117 ± 0.009) cohorts (*P* > 0.05) (Fig. 7). The models trained using the combined QTL and RNA-Seq feature sets generally improved prediction accuracy for all five training/validation population designs, but this appeared to be entirely due to the effects of the QTL feature selected markers (Fig. 7).

Models trained using the QTL feature selected variants significantly increased the accuracy of GP relative to the HD analyses when trained and validated in the CA (QTL: 0.217 ± 0.030) and Joint (QTL: 0.200 ± 0.014) cohorts (*P* < 0.05), but not in the NM (QTL: 0.121 ± 0.025) cohort (P > 0.05) (Fig. 7). When training was performed using the data for one state and validation occurred using the data for the other state, prediction accuracy became positive and was 0.108 when training occurred in the larger CA cohort and validation was performed in the NM cohort.

To evaluate the magnitude of the bias in these results that was created by the prior use of the data for QTL discovery, we performed a 5-fold cross validation in which the data were randomly partitioned into 5 approximately equal data subsets and 5 analyses were performed in which 4 subsets (80% of the available data) were used for QTL discovery and model training and the fifth subset (20% of the available data) was used for estimating the accuracy of GPs estimated for these animals. From these analyses, the average accuracy across replicates was 0.082 ± 0.023 for the combined CA and NM cohorts, 0.083 ± 0.026 in the CA cohort and 0.079 ± 0.037 in the NM cohort. However, on average, of the top 100 QTL regions identified in each replicate analysis, only 53.0 ± 5.0 were in common with the top 100 QTL regions identified in the analysis of the whole data set.

To more closely capture the QTL architecture found in the analysis of the whole dataset, we performed a second cross validation analysis in which the data were randomly partitioned into 135 approximately equal data subsets and 135 analyses were performed in which 134 subsets (99.3% of the available data) were used for QTL discovery and model training and the fifth subset (0.7% of the available data) was used for estimating the accuracy of GPs. From these analyses, the average accuracy across replicates was 0.073 ± 0.238 for the combined CA and NM cohorts, 0.091 ± 0.284 in the CA cohort and 0.090 ± 0.463 in the NM cohort. The standard errors reflect the fact that the correlation for each replicate was estimated using 21 or fewer data points. On average, of the top 100 QTL regions identified in each replicate analysis, 91.3 ± 2.6 were in common with the top 100 QTL regions identified in the analysis of the whole data set.

To calibrate the results of the two-fold cross validation reported in Fig. 7 with the 5-fold cross validation results generated for the QTL feature selection analyses, we also performed a 5-fold cross validation for the GPs estimated using the HD marker set. For the HD markers, GPs had accuracies of 0.128 ± 0.023 for the combined CA and NM cohorts, 0.149 ± 0.031 in the CA cohort and 0.158 ± 0.090 in the NM cohort. These accuracies did not differ to those for GPs produced using the HD assay in the same cohorts (*P* > 0.05).

#### Comparing feature sets

Surprisingly, only 8379 SNPs were in common between the QTL feature selected SNP set (1.6% of SNPs) and the RNA-Seq feature selected SNP set (2.7%). This may have been impacted by the relatively small window-size (transcript ±25 kb) selected for sampling SNPs in the vicinity of genes that were differentially regulated in the bronchial lymph node in response to pathogen challenge. However, as has been previously observed in human complex disease GWAS studies, the largest effect QTL are rarely found within transcripts and are much more frequently located in intergenic and upstream regions that are inferred to have regulatory effects. This is consistent with our results, which suggest that the largest effect BRD-risk QTL are generally not located within close proximity to genes involved in the immune response to pathogen challenge, suggesting that these QTL must have regulatory roles on gene expression. This, of course, does not preclude certain variants within these transcripts from underlying QTL, but these QTL have too small an effect to have been detected in the GWAS of 2703 animals.

Using a two-fold cross validation analysis, GPs based on variants identified in QTL regions had higher accuracies than those based on variants within genomic regions harbouring genes for which expression was perturbed by pathogen challenge and also based on the union of two feature sets (Fig. 7). However, the results of the 5-fold and 135-fold analyses reveal that these results are significantly biased and that the QTL feature model actually performed no better in each state than did the model based on HD data (Fig. 7).

## Discussion

### Association analyses

#### Refined signal

The QQ plots reveal that the sample size for this study was not adequate for QTL detection using either the HD data or the imputed WGS data (Fig. 4). This was further supported by the results of the 5-fold and 135-fold QTL feature selection cross validation analyses in which only 53 and 91.3% of the top 100 QTLs identified in the analysis of the whole data were identified in the analysis of subsets.

of 80 and 99.3% of the entire data. Marker imputation slightly exacerbated this issue, but variants that were strongly associated with risk of BRD were identified within genomic regions harbouring genes with functions that were plausibly immune related [4]. Analysis of the combined CA and NM cohorts likely also contributed to this problem due to phenotype divergence that increased the complexity of the underlying genetic architecture of the trait due to the expansion of the range of pathogens responsible for disease in the two states (Fig. 6).

Nevertheless, imputation of WGS variants facilitated the refinement of QTL mapping in these data. By including more markers in the analysis that could potentially be in strong linkage disequilibrium with the causative variants underlying QTL, the resolution of the boundaries for QTL regions [e.g., *CSMD1* and *INPP4B*; Fig. 3] appear to have been improved. While this approach is not by itself capable of inferring the causality of any associated variant, the biological relevance of genes, particularly those differentially expressed in response to pathogen infection suggests the utility of using imputed WGS data for refining the numbers of variants that potentially may be causal for their effects on risk of disease. Imputation also enabled the identification of relatively small regions, which could be targeted for further fine mapping using variants missing from the 1KBGP data and indels. This approach could also benefit from the identification of regulatory regions within the QTL regions and specifically targeting the genotyping of variants within regulatory regions located within QTL. These types of fine mapping analyses have been found to be useful in human GWAS studies [15].

#### Inconsistency between states

Imputation of WGS variation did not significantly improve the accuracies of GP in the analysis of the data from the combined state cohorts, almost certainly due to the differences in the genetic basis of susceptibility to BRD caused by different pathogens (Fig. 6) [4]. While the estimates of PVE increased using the imputed WGS variant data, PVE in the combined CA and NM cohorts was still lower than for either state. Furthermore, QTL were not, in general, common between the CA and NM cohorts (Fig. 5).

A key goal of research into the genetic architecture of risk of BRD must now be to differentiate universal from environmental- or pathogen-specific QTL. With larger sample sizes both within and across regions of the US, and more accurate imputation of WGS this may become feasible. High-resolution WGS QTL mapping will be also crucial for distinguishing between single QTL and multiple closely linked pathogen-specific QTL. We hypothesize that the largest effect BRD risk QTL found in the analysis of each cohort are primarily pathogen specific but that there may be more extensive QTL sharing among the loci with smaller effects.

### Genomic predictions

#### Regional differences in BRD phenotypes

Region-specific effects represent a key finding of our GP for BRD risk. We were able to train models that effectively predicted the risk of BRD when training occurred within each state. However, when we attempted to train and validate a model using data from both states, prediction accuracy declined despite an increase in the size of the training and validation populations. Typically, the accuracies of GP improve as training population size increases [16]. Perhaps more striking, was the inability using WGS imputed variation, to predict disease risk across states when training was restricted to data from a single state. This indicates that substantial impediments exist for the deployment of GP for risk of infectious disease caused by multiple pathogens under the current genomic selection paradigm [17].

Genomic prediction models generated in this study may perform poorly in environments where the pathogen profiles differ from those of the study environments (Fig. 6). Moreover, the ongoing collection of data that will be aggregated across environments for analysis, which typically ensures accurate predictions, will not solve this problem if susceptibility to different pathogens represents a set of traits, that are at best, moderately genetically correlated. Our estimate of a negative genetic correlation between the risk of BRD in two U.S. regions suggests that this may be the situation. Genomic prediction models typically exploit the abundance of phenotypic data that are available for non-genotyped animals to estimate the genetic merit of animals and build GP using either one- or two-step procedures [18, 19]. Unlike routinely recorded production traits, the aggregation of phenotypes from different environments to produce a single national evaluation of genetic merit for risk of BRD, may not be possible.

#### Causes of phenotypic heterogeneity

We investigated alternative explanations for the reduced utility of the pooled data from the CA and NM cohorts for GWAS and GP. We found no evidence of population substructure between the animals from the two states (Fig. 8) which agrees with the principal component analysis results reported by Neibergs et al. [4]. Moreover, the QQ plot for the GWAS of the data for the combined CA and NM cohorts does not suggest a structural effect that was absent in the GWAS for the individual states (Fig. 4). With the widespread use of artificial insemination in U.S. Holsteins and a primary emphasis nation-wide on a single breeding objective, Net Merit, population structure was not expected and GP should be translatable population-wide [17]. The possibility of a large systematic bias in phenotyping between the regions is also highly unlikely considering the objective nature of the McGuirk scoring system and the fact that these data were collected by trained veterinary personnel [4]. Pathogen diagnostic data obtained from mid-nasal and deep-nasopharyngeal swabs taken from each calf made it clear that there were very different pathogen profiles present in the two state cohorts, and consequently, disease status classification must be interpreted from the perspective of the pathogens to which the animals were exposed in each environment (Fig. 6).

Examination of the large effect QTL found in the analysis of data from one state cohort but not in the other provides some biological clues as to the roles of these QTL. *PPARG* was detected as a QTL in the analysis of the NM cohort, but was not among the 100 most significant QTL in either the CA or combined CA and NM cohort GWAS. *PPARG* is a transcription factor that has been shown to mediate immune responses in a pathogen-specific manner. In human Coronavirus respiratory infections, expression of *PPARG* is crucial for preventing a persistent infection [20]. Host *PPARG* variants have also been found to provide resistance to infection by some, but not all, Hepatitis C virus strains [21]. The allele frequencies at imputed WGS variants in the vicinity of *PPARG* (chr22:57,366,988-57,413,013 bp) were similar for the animals in the two state cohorts which would be expected if similar sires are selected nation-wide based upon their Net Merit PTA. However, only one of the 5 single pathogen challenges, *Mannheimia haemolytica*, resulted in the upregulation of expression of *PPARG* in the bronchial lymph nodes of experimentally challenged cattle [7] suggesting a pathogen-specific role for *PPARG* in response to BRD.

A small number of QTL were found in common in the analyses of both state cohorts. Some of these QTL may be due to common pathogens, but they may also potentially reflect shared immunological risk factors that are not pathogen specific. Identification of these variants may be vital to the development of a single nation-wide GP for risk of BRD and these QTL should be discoverable in the analysis of data pooled across geographical regions. However, selection to reduce the capacity of pathogens to infect their host may trigger an evolutionary arms race between pathogens and hosts as predicted by the red queen hypothesis, which states that rapid evolution and high genetic diversity is favoured in competitive environments [22]. However, focusing selection on small-effect variants that improve resistance to a broad spectrum of pathogens should not result in strong selection for increased pathogen virulence but should allow reductions in BRD prevalence nation-wide.

#### Feature selection

We utilized feature selection to train GP models using only genetic variants that were potentially more likely to be associated with disease susceptibility than randomly selected variants. This was accomplished by selecting variants from regions of the genome that had been identified as associated with risk of BRD in the combined CA and NM cohort GWAS and also regions containing genes for which expression in the bronchial lymph node was perturbed in animals experimentally challenged with single pathogens that cause BRD. Our intention was to enrich the prediction model for variants that were more likely to be trait associated and to reduce the noise in the prediction model created by the estimation of a multitude of SNP effects for variants that are not trait associated. Feature selection reduced the number of variants used in prediction by an order of magnitude relative to the imputed WGS training data, and to a level comparable to the HD training data. The GWAS for the individual state and combined CA and NM cohorts revealed BRD risk QTL segregating in both cohorts despite the differences in pathogen profiles in the two environments (Fig. 6). We also anticipated that smaller effect QTL might reliably be captured by including variants located in the genomic regions harbouring genes for which expression was perturbed in the normal immune response to BRD pathogens [7].

In a replicated two-fold cross validation analysis, the RNA-Seq feature selected set performed no better than the model that used the HD variants suggesting that cis-acting variants within genes involved in the normal immune response to pathogens responsible for BRD do not significantly impact risk of BRD. The identification of trans-acting loci such as transcription factors that control the regulation of expression of these genes might therefore be a more valuable approach for feature selection. Moreover, improvement in prediction accuracy using the feature selection model that combined variants located in both the QTL and RNA-Seq feature selected variant sets appears to primarily have been driven by the overestimated accuracy increases from QTL feature selection (Fig. 7).

The accuracy of GP trained in one half of the combined CA and NM cohorts and validated in the other half of the cohorts using the QTL feature selected variants was estimated to increase by 73% over the accuracy achieved using the HD data. However, when we implemented 5-fold and 135-fold cross validation analyses to remove the data for animals for which GPs were to be produced from the QTL identification and training model analyses, the accuracies of the produced GPs did not differ from those using the HD markers in the two-fold cross validation analyses (*P* > 0.05). This was not due to a difference in calibration between the analyses. Accuracies of GPs produced using the HD assay did not differ (*P* > 0.05) for any of the three cohorts using the two-fold or 5-fold cross validation analyses. From this, we conclude that the GP accuracies produced by the two-fold cross validation for the feature selection sets are significantly biased upward by the a priori use of the data to identify QTL regions. However, the produced GPs do not appear to be biased. Eliminating the analyses that involved training using the data for one state and validating in the other (where the genetic correlation between BRD risk was − 0.36), the mean regression of GP on observed BRD phenotype was 1.04 (Additional file 1 Table S1).

We anticipated that the use of variants located in QTL regions identified in the joint analysis of the CA and NM cohorts would generate GPs based upon immunological risk variants with common directional effects on the risk of BRD across the two state environments. While the analyses based on these markers produced strongly biased GP accuracies within each of the states, accuracies of the GPs trained in one state and validated in the other became positive using the QTL feature selected variants, despite the negative genetic correlation between risk of BRD in the two states. While the sample size of 2703 individuals is insufficient for the accurate identification of QTL within and across the CA and NM cohorts and also for evaluating the QTL feature selection approach, this result merits additional consideration. Further research should investigate how to most successfully model the physiology of the disease and incorporate variation within biologically relevant pathways into the prediction model [23].

### Implications for future breeding

Environments other than those sampled in this study could differ significantly for their pathogen profiles or the pathogen profile within each environment may vary significantly across years. If the regional pathogen profiles tend to be stable in their composition across time, it may be most effective to develop region-specific BRD risk predictions, and associate these with specific regional environmental conditions [23]. Alternatively, a national genetic evaluation could focus selection on the variants that influencing risk of disease in a pathogen agnostic manner. The feature selection approaches implemented here targeted the latter goal, but did not specifically attempt to functionally validate or elucidate the biology associated with variation within the selected genomic regions. For example, a QTL may be specific to an individual pathogen that is present in both regional cohorts or may be specific to all bacterial or viral pathogens that cause BRD. This level of refinement in our understanding of BRD will require considerably more data aggregation, improved mapping resolution and biological experimentation. Selection without regard to these potential GxE effects will be effective only if the heritability of disease risk across all environments is moderate or the pathogen profiles within specific regions of the country remain stable in time. Indeed, this may explain why BRD has been persistent despite the considerable cost associated with animal infection.

### Imputation

In both empirical and theoretical studies, others have demonstrated limited improvements in prediction accuracy using imputed WGS variation [24]. Studies have also shown that improvements in prediction accuracy are greatest in validation populations that are more distantly related to the training population [8, 25]. The primary challenges for genomic prediction based on imputed WGS variation are imputation accuracy and informed feature selection approaches to select the variants that should be included in the prediction model.

The potential benefit of high accuracy imputation lies in capturing genetic variation that is missed due to low LD with the common variants on the currently available genotyping assays. Given the abundance of rare variation in the cattle genome, many functional variants will not be in strong LD with these common variants. Reliance on LD and allele phase relationships between chip-based markers and causal variants, which are population specific, explains why GP models frequently fail to be translatable across populations [16]. Accurate WGS imputation may address this issue, however, this appears to be difficult to accomplish if we the imputation process requires strong LD between typed and untyped markers [26]. However, current imputation algorithms generally exploit a haplotype matching strategy which can accurately impute common haplotypes. Despite the relatively small effective population size, there is abundant variation within Holsteins. However, this variation resides on a relatively small number of haplotypes which enables the majority of common haplotypes to be captured by sequencing only a few hundred animals [6].

We anticipate that incrementally less expensive and higher quality sequence data will result in many more sequenced individuals in the near future. In conjunction with processing these data against considerably improved reference assemblies, this will enable the accurate prediction of increasingly rare haplotypes and will enable the detection of identity by descent between haplotypes in the sequenced training population and imputed population individuals. Strategically selecting individuals for genotyping and phenotyping that can be accurately imputed to WGS, such as the direct descendants of sequenced sires appears to be a very useful strategy [27–30]. Under these circumstances, the best imputation methodologies can achieve imputation accuracies of 99% across the full spectrum of allele frequencies [29].

### Assembly issues and genomic complexity

Imputation accuracy for variants predicted to reside in poorly assembled regions was limited in this study. This was evidenced by large regional decreases in imputation accuracy (Fig. 1) as well as the challenge in correctly calling indels which were eliminated from this study. Cattle genomes have an abundance of structural, repeat and indel variation [31, 32]. We expect that improvements in the reference genome assembly will soon lead to substantial benefits for variant calling and the physical ordering of loci within haplotypes. Assemblies created using long read technologies, chromatin capture and optical maps possess great improvements in contiguity [33]. As the cost of sequencing continues to decrease, genotyping will be enabled by long read or synthetic long read technologies which can detect structural variation, directly phase haplotypes, and also enable individual-specific sequence assembly [34].

### Imputed variant selection for prediction models

We currently have the capability to impute the large numbers of variants that have been identified in recent sequencing efforts at moderate to high accuracies. However, the accuracy and large number of called WGS variants and relatively small number of sequenced individuals all impact the accuracy of imputation. One approach towards the amelioration of this issue is to assign annotations to classes of variants, and then fit classes separately in a mixture model of normal distributions with different scale parameters. If a class, such as variants within transcripts found to be trait-associated in an RNA-Seq study, is enriched for alleles that directly cause trait variation, the power to detect these causal variants should be improved by increasing the prior likelihood of association for members of this class. Such an approach is implemented in BayesRC [8]. To the extent that annotations and genotype imputation can be accomplished at high levels of accuracy, this approach should be beneficial for variant selection for GP. However, at present, both variant annotation and imputation are limited in extent and accuracy, and rather than restricting inclusion to specific variants, we defined sets of genomic regions (based on windows of SNPs) of interest and included all variants within these windows. Once the windows had been defined, the Bayesian Sparse Linear Mixed Model (BSLMM) [35] implemented in GEMMA had no additional information about which of the variants within each window may be important. BSLMM assumes that only a small proportion of the supplied variants have large effects that contribute to variation in the phenotype and estimates which of the supplied variants have large effects, the magnitudes of these effects and the residual additive genetic merit of each individual due to the remaining small effect variants. Within each feature selected window, if a causal variant acted by disrupting an enhancer, for example, BSLMM would be expected to identify this variant as a large effect variant. BSLMM would also identify other loci in strong LD with the causal variant and sampling effects could lead to some of these having stronger associations than the causal variant.

## Conclusions

We investigated approaches to generate GP for risk of BRD across two geographic regions of the U.S. that appear to differ for their pathogen profiles. While the animals were all phenotyped using a common robust evaluation protocol [2], the genetic architecture of the trait differed profoundly between the two U.S. states. We hypothesized that imputation of HD genotypes to WGS variants in conjunction with biologically guided feature selection may enable the development of genomic prediction models in Holstein cattle with utility across geographic regions. While the genome sequence resources for WGS imputation and the annotation of identified genomic variants in cattle are limited, the continued improvement of these resources should enable this development. The use of the BSLMM applied to feature selected variants for genomic prediction reduces the need for assigning annotations or identifying causality when utilizing millions of variants to model a complex biological phenotype.

Further work is required to investigate the pathogen-specific genetic architectures of susceptibility to BRD and the extent to which pathogen profiles differ spatially and temporally nationwide to enable the development of GP models that will reduce disease prevalence nationally. This will require an extensive ongoing effort to capture animal phenotypes and genotypes and to assess pathogen profiles nationwide. Selection to reduce BRD prevalence will require a better understanding of the genetic architecture of resistance to specific pathogens, the magnitudes of genetic correlations between pathogen-specific BRD phenotypes, casual variant discovery and improved genomic resources for imputation and variant annotation.

## Methods

### Data collection

Whole blood samples were collected from a commercial calf raising facility in CA and from commercial Holstein dairy herds in NM as described by Neibergs et al. [4]. BRD cases and controls were sampled by first identifying a clinically ill animal and then selecting a nearby contemporary animal without signs of illness. Calves were scored for five BRD indicator traits using the McGuirk Scoring system [2, 4]. Controls were followed in time to ensure that they did not develop illness after the initial observation. Extracted DNA samples from all animals were assayed with the Illumina BovineHD assay. Variants from both the CA and NM cohorts were filtered on allele frequency and per animal call rate, resulting in 654,044 loci available for analysis. After final filtering on phenotype availability, 2703 samples were available, with 1978 and 725 being from CA and NM, respectively. A complete description of the sampling, phenotyping and genotyping processes was provided in Neibergs et al. [4].

### Anesthetic and euthanasia methods

The experimental animals were not anesthetized or euthanized in order to conduct this study [4].

### Imputation pipeline

#### A/B to ref/alt

The first step in genotype imputation required converting Illumina A/B allele calls to reference assembly sequence variant and alternate alleles. The HD variant genotypes were converted from A/B allele calls to reference/alternate (ref/alt) alleles in an allelic dose format (0 for homozygous for the reference assembly allele, 1 for heterozygote, and 2 for homozygous for the alternate allele). This conversion process was accomplished using an analysis of 94 animals that had been both whole genome sequenced and also genotyped with the Illumina BovineHD or BovineSNP50 genotyping platforms. This empirical verification of the SNP allele identities was necessary as the BovineSNP50 manifest (which was carried across to the manifest for the HD assay) was found to contain a very high percentage of inconsistent A/B to ref./alt allele mappings. These inconsistencies appear to have been caused by changes in strand orientations within the reference assembly between the time of the design of the BovineSNP50 assay and the release of the UMD3.1 reference assembly. This was described in greater detail in Taylor et al. [36].

#### Reference genome sequences

The reference set of haplotyped WGS variants used for imputation was the Run5 data from the 1KBGP, which included 39,721,988 variants phased and imputed in 1578 animals [6]. The Run5 data set included WGS for 450 Holstein animals, to which we added WGS for 29 Holstein animals sampled from the BRD GWAS study population [4]. These animals were sequenced to an average depth of 11.94X and variants were called as described in Taylor et al. [36]. The sites included in the imputation reference set included only the SNP variants found in the Run5 1KBGP data. We removed all indels from the Run5 1KBGP data, due to previously identified issues with Samtools indel calling [37]. High indel genotyping error rates can significantly impact phasing and genotype imputation quality; particularly with imputation algorithms such as FImpute [38] that do not explicitly attempt to model genotype errors. Only genotypes for the SNPs identified within the Run5 1KBGP data were extracted from the variant calls for our 29 independently sequenced Holsteins. Phasing and imputation of these data were performed using FImpute.

### Filtering

We filtered every variant that had an allele frequency < 5% or a genotype variance < 0.1% in the WGS imputed data set for the 2703 Holsteins. A separate imputation run in which the 29 sequenced Holsteins were excluded from the reference animal set was used to assess imputation accuracy. The genotypes imputed for these 29 animals from their HD genotypes were correlated with the genotypes independently called from their WGS after converting both to dosage format as previously described. The resulting set of site-specific imputation accuracies (correlations between imputed and WGS-called genotypes) was then used to filter individual variants that had accuracies of < 65%. The 95% confidence interval for a correlation coefficient of 65% based on 29 samples is from 37 to 82% and while this threshold will include variants with low imputation accuracy, we wished to avoid filtering variants that were imputed with a reasonably high accuracy.

### Imputation accuracy and reference assembly quality

Many variants included in the 1KGBP imputation reference sequence set were not observed in our 29 sequenced animals and the quality of genotype imputation could not be assessed for these loci. Consequently, we decided to classify the accuracy of imputation by variably sized regions of the genome to identify those regions for which genotype imputation could not be accurately accomplished due to local misassemblies or misoriented contigs within the reference assembly. To accomplish this, we applied GenWin to the SNP genotype imputation accuracy correlation estimates to identify genomic regions for which SNP genotypes were imputed with consistently low accuracy. Windows were analysed using an initial window size of 10 kb and GenWin pooled adjacent windows for which imputation accuracies were similar. All variants within windows for which the average imputation accuracy was < 65% were removed from the data (Fig. 1) including SNPs that were not detected in the 29 sequenced animals. Less than 30% of the SNPs had direct estimates of their imputation accuracy either because the sample of 29 sequenced animals was not sufficient to capture the majority of the variants segregating in Holsteins, or because the multibreed reference panel resulted in the imputation of genotypes for variants that were not segregating in Holsteins.

### Association analysis

#### Model

Association analysis was performed in GEMMA using a univariate linear mixed models [39]. Fixed effects of sex and age were included as covariates within the analyses of the binary case-control disease phenotype data for each state, and a fixed effect for state was included in the model for the analysis of the combined CA + NM data. The model also included a random effect for the additive genetic merit of each animal and a random residual term that contained residual additive genetic effects that are not in complete linkage disequilibrium with the markers included in the model [40] and non-additive genetic, permanent and temporary environmental effects. The GRM was constructed using a random sample of 10% (*N* = 928,258) of the genomic sites represented in the imputed WGS genotype data approximately consistent with number of SNPs present on the HD assay. For the analyses of the HD genotype data all SNPs were used to form the GRM. We also estimated the genetic correlation between the binary case-control BRD variables in CA and NM using GCTA by fitting a bivariate model.

#### Genomic predictions

Genomic predictions for BRD risk were obtained using the GEMMA implementation of the BSLMM. Training and validation were performed using cross-validation with five data subsets. Three training and validation sets were produced by randomly sampling one half of the animals within the CA, NM or combined CA + NM cohorts and training was performed in one half and validation in the other half of the data. This process was repeated five times to enable the estimation of sampling variances of the GP accuracies for each animal cohort and analysed marker set. Training using the CA data with validation performed using the NM data and vice versa was also performed.

### Feature selection

#### QTL

Feature selection was used to construct GP using subsets of the imputed WGS variants that resided within genomic regions of biological relevance. The QTL-associated variants were selected from QTL windows identified in the univariate association analyses. We again utilized GenWin to identify the size of each QTL window, by analyzing the –log_10_P-values for the Wald association test for SNPs tagging each QTL. QTL windows were initially set to a size of 500 kb. The most significant 100 QTL identified in each analysis were used in each feature selection analysis, with window boundaries determined by GenWin. All of the variants within each of these QTL windows were used to train the genomic prediction models.

#### RNA-SEQ

WGS imputed variants were selected for inclusion in the prediction model based on their proximity to genes identified as being differentially expressed in the bronchial lymph nodes of non-challenged control animals and animals that had been artificially challenged with single pathogens causing BRD in a challenge experiment performed in beef cattle and reported by Gerschwin et al. [41]. The RNA-Seq data were processed using the Tuxedo suite, as described in the original study [7, 42]. The 1000 genes (~ 5% of all annotated bovine genes) with the most significant expression changes between controls and challenged animals were selected, and all variants within the region from 25 kb upstream to 25 kb downstream of each gene transcript were included in the GP analyses.

### Genomic predictions

The models based on the RNA-Seq and QTL selected features used the same GRM as the WGS model and were analysed using GEMMA’s BSLMM. The total numbers of variants within each feature selection class were: 9,282,726 for imputed WGS, 654,044 for the HD, 529,942 for QTL regions, 310,782 for RNA-Seq differentially expressed gene regions and 832,345 for the union of the QTL and RNA-Seq identified regions. All GPs were correlated with the binary phenotypes for the individuals in the validation set to assess the accuracy of the predictions.

## Additional file

Additional file 1: **Table S1.** Prediction accuracies and regressions of phenotype on genomic predictions for risk of BRD according to training and validation phenotype subsets and genotype subsets. This file contains Pearson correlation coefficients, regression slopes and intercepts for the regression of BRD case/control phenotype on genomic predictions for BRD risk for the Bovine HD, WGS imputed and feature selection genotype sets. (XLSX 10 kb)

## Data Availability

Genotype and phenotype data are available from CS at Texas A&M University for non-commercial use following the execution of a Materials Transfer Agreement. RNA-Seq data are available at the National Center for Biotechnology Information Sequence Read Archive under accession number SRP052314.

## Abbreviations

1KBGP: 1000 Bull Genomes Project
BRD: Bovine respiratory disease
BSLMM: Bayesian Sparse Linear Mixed Model
CA: California
GP: Genomic predictions
GRM: Genomic relationship matrix
GWAS: Genome-wide association study
HD: BovineHD
NM: New Mexico
PVE: percent of variation explained
QTL: Quantitative Trait Locus
RNA-Seq: Next generation messenger RNA transcriptome sequencing
SNP: Single nucleotide polymorphism
WGS: Whole genome sequence

## References

[1] Neibergs HL, Neibergs JS, Wojtowicz AJ, Taylor JF, Seabury CM, Womack JE (2014). Economic benefits of using genetic selection to reduce the prevalence of bovine respiratory disease complex in beef feedlot cattle. Proceedings of the 2014 beef improvement federation annual meeting and convention.

[2] McGuirk SM (2008). Disease management of dairy calves and heifers. Vet Clin North Am Food Anim Pract.

[3] Cole JB, VanRaden PM (2018). Possibilities in an age of genomics: the future of selection indices. J Dairy Sci.

[4] Neibergs HL, Seabury CM, Wojtowicz AJ, Wang Z, Scraggs E, Kiser J (2014). Susceptibility loci revealed for bovine respiratory disease complex in pre-weaned Holstein calves. BMC Genomics.

[5] VanRaden PM (2008). Efficient methods to compute genomic predictions. J Dairy Sci.

[6] Daetwyler HD, Capitan A, Pausch H, Stothard P, van Binsbergen R, Brøndum RF (2014). Whole-genome sequencing of 234 bulls facilitates mapping of monogenic and complex traits in cattle. Nat Genet.

[7] Tizioto PC, Kim J, Seabury CM, Schnabel RD, Gershwin LJ, Van Eenennaam AL (2015). Immunological response to single pathogen challenge with agents of the bovine respiratory disease complex: an RNA-sequence analysis of the bronchial lymph node transcriptome. PLoS One.

[8] MacLeod IM, Bowman PJ, Vander Jagt CJ, Haile-Mariam M, Kemper KE, Chamberlain AJ (2016). Exploiting biological priors and sequence variants enhances QTL discovery and genomic prediction of complex traits. BMC Genomics.

[9] Edwards SM, Sørensen IF, Sarup P, Mackay TF, Sørensen P (2016). Genomic prediction for quantitative traits is improved by mapping variants to gene ontology categories in Drosophila melanogaster. Genetics.

[10] Sarup P, Jensen J, Ostersen T, Henryon M, Sørensen P (2016). Increased prediction accuracy using a genomic feature model including prior information on quantitative trait locus regions in purebred Danish Duroc pigs. BMC Genet.

[11] Veerkamp RF, Bouwman AC, Schrooten C, Calus MP (2016). Genomic prediction using preselected DNA variants from a GWAS with whole-genome sequence data in Holstein–Friesian cattle. Genet Sel Evol.

[12] Xu S (2003). Theoretical basis of the Beavis effect. Genetics.

[13] Beissinger TM, Rosa GJ, Kaeppler SM, Gianola D, de Leon N (2015). Defining window-boundaries for genomic analyses using smoothing spline techniques. Genet Sel Evol.

[14] Lee SH, Yang J, Goddard ME, Visscher PM, Wray NR (2012). Estimation of pleiotropy between complex diseases using single-nucleotide polymorphism-derived genomic relationships and restricted maximum likelihood. Bioinformatics.

[15] Claussnitzer M, Dankel SN, Kim KH, Quon G, Meuleman W, Haugen C (2015). FTO obesity variant circuitry and adipocyte browning in humans. N Engl J Med.

[16] Habier D, Fernando RL, Garrick DJ (2013). Genomic BLUP decoded: a look into the black box of genomic prediction. Genetics.

[17] García-Ruiz A, Cole JB, VanRaden PM, Wiggans GR, Ruiz-López FJ, Van Tassell CP (2016). Changes in genetic selection differentials and generation intervals in US Holstein dairy cattle as a result of genomic selection. Proc Natl Acad Sci U S A.

[18] Legarra A, Aguilar I, Misztal I, Christensen OF (2014). Single step, a general approach for genomic selection. Livest Sci.

[19] Garrick DJ, Taylor JF, Fernando RL (2009). Deregressing estimated breeding values and weighting information for genomic regression analyses. Genet Sel Evol.

[20] Al-Qahtani AA, Lyroni K, Aznaourova M, Tseliou M, Al-Anazi MR, Al-Ahdal MN (2017). Middle east respiratory syndrome corona virus spike glycoprotein suppresses macrophage responses via DPP4-mediated induction of IRAK-M and PPARγ. Oncotarget.

[21] Cai T, Dufour JF, Muellhaupt B, Gerlach T, Heim M, Moradpour D (2011). Viral genotype-specific role of PNPLA3, PPARG, MTTP, and IL28B in hepatitis C virus-associated steatosis. J Hepatol.

[22] Van Valen L (1973). A new evolutionary law. Evol Theory.

[23] Technow F, Messina CD, Totir LR, Cooper M (2015). Integrating crop growth models with whole genome prediction through approximate bayesian computation. PLoS One.

[24] Pérez-Enciso M, Rincón JC, Legarra A (2015). Sequence- vs. chip-assisted genomic selection: accurate biological information is advised. Genet Sel Evol.

[25] Lu D, Akanno EC, Crowley JJ, Schenkel F, Li H, De Pauw M (2016). Accuracy of genomic predictions for feed efficiency traits of beef cattle using 50K and imputed HD genotypes. J Anim Sci.

[26] Snelling WM, Kuehn LA, Keel BN, Thallman RM, Bennett GL (2017). Linkage disequilibrium among commonly genotyped SNP variants detected from bull sequence. Anim Genet.

[27] Gorjanc G, Cleveland MA, Houston RD, Hickey JM (2015). Potential of genotyping-by-sequencing for genomic selection in livestock populations. Genet Sel Evol.

[28] Livne OE, Han L, Alkorta-Aranburu G, Wentworth-Sheilds W, Abney M, Ober C (2015). PRIMAL: fast and accurate pedigree-based imputation from sequence data in a founder population. PLoS Comput Biol.

[29] Steinthorsdottir V, Thorleifsson G, Sulem P, Helgason H, Grarup N, Sigurdsson A (2014). Identification of low-frequency and rare sequence variants associated with elevated or reduced risk of type 2 diabetes. Nat Genet.

[30] Bickhart DM, Hutchison JL, Null DJ, VanRaden PM, Cole JB (2016). Reducing animal sequencing redundancy by preferentially selecting animals with low-frequency haplotypes. J Dairy Sci.

[31] Bickhart DM, Hou Y, Schroeder SG, Alkan C, Cardone MF, Matukumalli LK (2012). Copy number variation of individual cattle genomes using next-generation sequencing. Genome Res.

[32] Xu L, Haasl RJ, Sun J, Zhou Y, Bickhart DM, Li J (2017). Systematic profiling of short tandem repeats in the cattle genome. Genome Biol Evol.

[33] Bickhart DM, Rosen BD, Koren S, Sayre BL, Hastie AR, Chan S (2017). Single-molecule sequencing and chromatin conformation capture enable de novo reference assembly of the domestic goat genome. Nat Genet.

[34] Weisenfeld NI, Kumar V, Shah P, Church DM, Jaffe DB (2017). Direct determination of diploid genome sequences. Genome Res.

[35] Zhou X, Carbonetto P, Stephens M (2013). Polygenic modeling with bayesian sparse linear mixed models. PLoS Genet.

[36] Taylor JF, Whitacre LK, Hoff JL, Tizioto PC, Kim JW, Decker JE (2016). Lessons for livestock genomics from genome and transcriptome sequencing in cattle and other mammals. Genet Sel Evol.

[37] Hwang S, Kim E, Lee I, Marcotte EM (2015). Systematic comparison of variant calling pipelines using gold standard personal exome variants. Sci Rep.

[38] Sargolzaei M, Chesnais JP, Schenkel FS (2014). A new approach for efficient genotype imputation using information from relatives. BMC Genomics.

[39] Zhou X, Stephens M (2012). Genome-wide efficient mixed-model analysis for association studies. Nat Genet.

[40] Taylor JF (2014). Implementation and accuracy of genomic selection. Aquaculture..

[41] Gershwin LJ, Van Eenennaam AL, Anderson ML, McEligot HA, Shao MX, Toaff-Rosenstein R (2015). Single pathogen challenge with agents of the bovine respiratory disease complex. PLoS One.

[42] Trapnell C, Roberts A, Goff L, Pertea G, Kim D, Kelley DR (2012). Differential gene and transcript expression analysis of RNA-seq experiments with TopHat and cufflinks. Nat Protoc.

